# Breed-specific immune regulation under endemic exposure to *Leishmania infantum* and other vector-borne pathogens in a native Mediterranean canine population

**DOI:** 10.1186/s13071-026-07337-w

**Published:** 2026-03-09

**Authors:** Lola Martínez-Sáez, Pablo Jesús Marín-García, Raffaella Cocco, Luigi Liotta, Lola Llobat

**Affiliations:** 1 Molecular Mechanisms of Zoonotic Diseases (MMOPS) Research Group, Department of Animal Production and Health, Public Veterinary Health and Food Science and Technology, School of Veterinary Medicine, Universidad Cardenal Herrera-CEU, CEU Universities, 46113 Valencia, Spain; 2 Department of Animal Production and Health, Public Veterinary Health and Food Science and Technology, School of Veterinary Medicine, Universidad Cardenal Herrera-CEU, CEU Universities, 46113 Valencia, Spain; 3 Department of Veterinary Sciences, Teaching Veterinary Hospital, 07100 Sassari, Italy; 4 Department of Veterinary Sciences, University of Messina, 98168 Messina, Italy

**Keywords:** Cytokines, Fonni dog, Immune tolerance, *Leishmania*, Mediterranean breeds

## Abstract

**Background:**

Canine leishmaniosis caused by *Leishmania infantum* remains a major zoonotic concern in the Mediterranean basin, where native breeds may have evolved adaptive immune mechanisms under long-term endemic exposure. The Fonni dog, indigenous to Sardinia, may represent a model of such adaptation. This study aimed to compare cytokine and growth factor profiles between Fonni dogs and German Shepherd dogs exposed to *L. infantum* and other vector-borne pathogens, to investigate potential breed-associated immune regulatory patterns.

**Methods:**

Fifty-nine clinically healthy dogs (Fonni and German Shepherds) living in endemic areas were included. Serum samples were tested for antibodies against *Anaplasma phagocytophilum, Ehrlichia canis, Leptospira spp., Leishmania infantum, and Rickettsia* spp. Concentrations of eleven cytokines and growth factors were measured using a multiplex bead-based immunoassay. Statistical analyses evaluated differences between breeds, associations with serological status and age, as well as correlation matrices and principal component analysis to explore clustering patterns among immune mediators.

**Results:**

Fonni dogs showed significantly higher serum concentrations of IL-10, NGF-β, IFN-γ, TNF-α, and VEGF-α compared with German Shepherds. Seropositive dogs for L. infantum and Rickettsia spp. exhibited increased levels of IL-10, NGF-β, and TNF-α. Age influenced cytokine expression, with young Fonni dogs displaying the highest IL-10 and NGF-β values, whereas TNF-α and MCP-1 concentrations increased with age. Correlation and principal component analyses revealed distinct breed-specific clustering, highlighting coordinated regulation of pro-inflammatory and angiogenic mediators, particularly IL-6, TNF-α, MCP-1, and VEGF-α.

**Conclusions:**

The elevated levels of several cytokines and growth factors in Fonni dogs suggest a breed-associated immune phenotype characterized by a regulatory/pro-inflammatory balance consistent with a tolerance-like response under endemic exposure. These findings support the hypothesis that native breeds such as the Fonni dog may have developed adaptive immunological mechanisms that limit immunopathology while maintaining effective responses in areas endemic for canine vector-borne pathogens.

**Graphical abstract:**

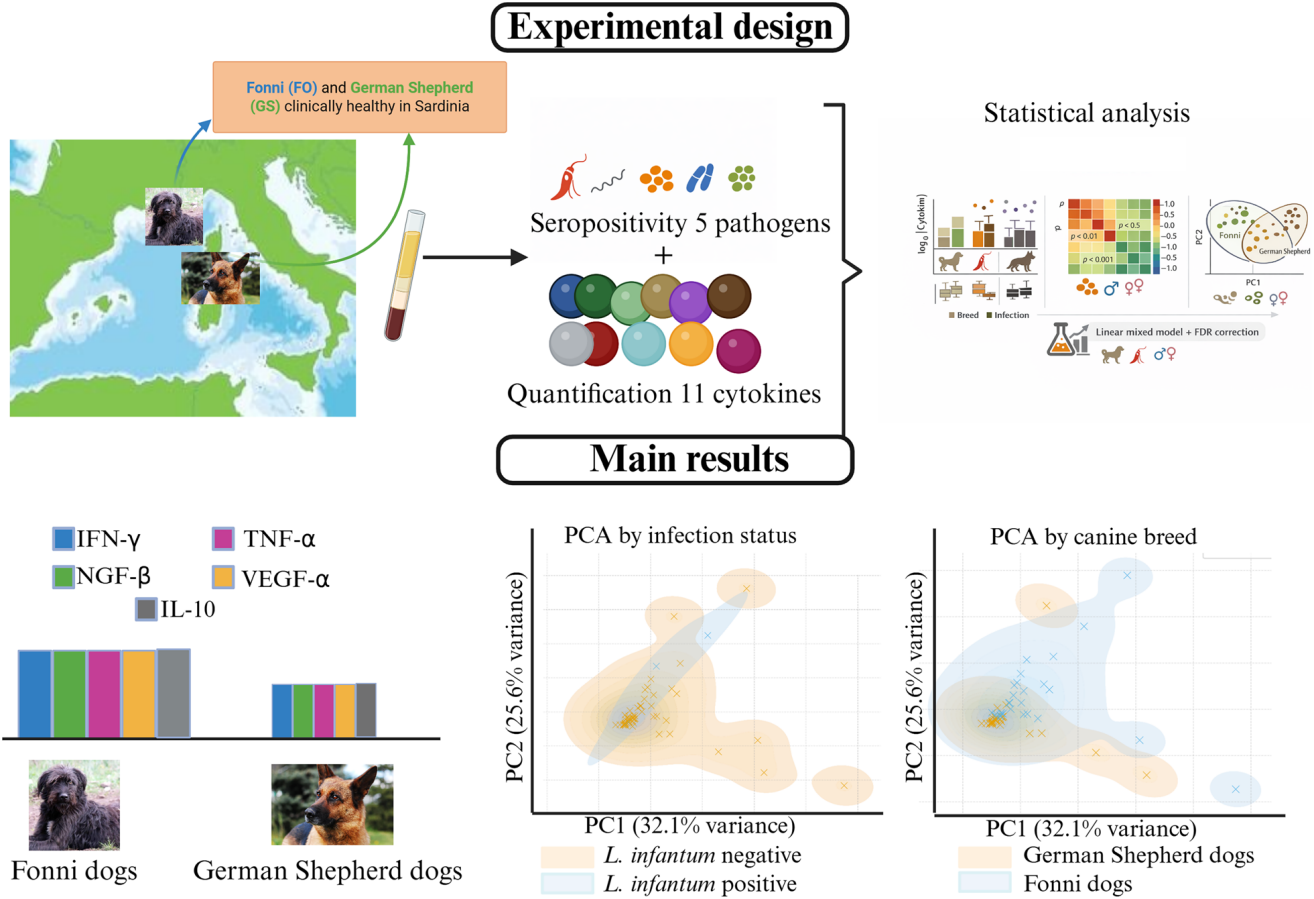

## Background

Vector-borne diseases (VBDs) are among the most significant infectious threats to domestic dogs worldwide, particularly in the Mediterranean basin, where climatic conditions favor the proliferation of phlebotomine sandflies, ticks, and fleas that transmit protozoal, bacterial, and rickettsial agents [1, 2]. Among these pathogens, *Leishmania infantum* is the etiological agent of zoonotic visceral leishmaniasis [3], a disease endemic to Africa, Asia, the Americas, and Europe [4]. Leishmaniasis has been endemic in the Mediterranean basin since antiquity, with historical, paleopathological, and epidemiological evidence supporting long-standing autochthonous transmission of *L. infantum* sustained by phlebotomine sandflies and canine reservoirs across southern Europe and the wider Mediterranean region [5–9]. Within Europe, visceral leishmaniasis persists in the Mediterranean region, particularly in Spain and Italy [7, 8], where the domestic dog (*Canis lupus familiaris*) serves as the primary reservoir host [10].

However, dogs in these regions are often exposed not only to *L. infantum* but also to other pathogens such as *Anaplasma phagocytophilum*, *Ehrlichia canis*, *Leptospira* spp., and *Rickettsia* spp., which may circulate simultaneously in overlapping ecological niches [11–14]. Co-infections or sequential exposures to multiple VBDs can modulate host immunity, potentially affecting both susceptibility to *L. infantum* and the clinical outcome of infection [15, 16]. The immunopathogenesis of canine leishmaniosis depends largely on the host’s ability to mount a protective Th1 immune response, characterized by interferon (IFN)-γ, interleukin (IL)-2, and IL-12 production, while disease progression is often associated with elevated IL-10 and other cytokines related to anti-inflammatory and angiogenic activity [17–20].

Interestingly, some Mediterranean dog breeds show a natural resistance to *L. infantum* infection. For instance, the Ibizan hound and Cirneco dell’Etna display enhanced pro-inflammatory cytokine profiles and genetic polymorphisms in immune-related genes such as *IFNG* and *IL6R*, which appear to confer partial protection against infection [21, 22]. This genetic and immunological diversity suggests that host factors play a critical role in disease progression. Nonetheless, little is known about other native Mediterranean breeds, including Fonni’s dogs (*Cane Fonnese*), a rustic Sardinian molossoid breed recognized by the Italian Kennel Club (ENCI). Archaeological evidence suggests its origin dates to the Bronze Age, highlighting its long-standing adaptation to the Mediterranean pathogens [14, 23].

Sardinia represents a unique epidemiological setting, where *L. infantum* coexists with a high prevalence of other vector-borne pathogens [24, 25]. In such ecosystems, assessing the immune responses of local breeds may offer valuable insights into the mechanisms underlying natural resistance to infection.

In this context, we investigated whether a native Mediterranean canine population chronically exposed to multiple vector-borne pathogens displays an immune profile consistent with tolerance to *L. infantum*, using the Fonni’s dog as a naturally adapted host and the German Shepherd as a comparative breed.

## Methods

This study is reported in accordance with the ARRIVE guidelines for animal research, including detailed descriptions of animal characteristics, inclusion and exclusion criteria, housing conditions, and statistical methods [26].

### Study population and sampling

A cross-sectional observational study was conducted between September 2024 and April 2025 in Sardinia (Italy), a Mediterranean island endemic for *L. infantum* and other canine vector-borne pathogens. In total, 59 privately owned, clinically healthy domestic dogs were included. Dogs were recruited consecutively during routine veterinary clinical visits, and the sample size corresponded to all animals meeting the inclusion criteria during the study period; therefore, no formal a priori sample size calculation was performed.

Clinical evaluation was performed by licensed veterinarians at the time of sampling. The absence of clinical signs compatible with canine leishmaniosis was assessed according to the LeishVet international guidelines, including lymphadenomegaly, weight loss, dermatological lesions (alopecia, exfoliative dermatitis, ulcers), ocular abnormalities, epistaxis, onychogryphosis, lethargy, and signs suggestive of renal involvement [27]. Dogs presenting clinical signs compatible with other systemic infectious diseases (e.g., fever, generalized lymphadenomegaly, mucosal pallor, dehydration, jaundice, or altered general condition) were excluded on the basis of routine physical examination. All owners provided written informed consent before inclusion.

Epidemiological data recorded for each animal included sex (male/female), age, and breed. Dogs were grouped by age into four categories: puppies (2–12 months), young (1–2 years), adults (3–8 years), and elder (9–11 years). Only dogs with documented pedigree certification were included in the breed analysis, which comprised the Fonni’s dog (*Cane Fonnese*) and German Shepherds.

Dogs were maintained under typical household conditions according to owner routines, with ad libitum access to food and water. Information on antiparasitic prophylaxis was obtained from owners and clinical records. None of the enrolled dogs had received topical pyrethroid-based repellents or insecticidal collars in the 12 months preceding sampling. No animal had received immunosuppressive therapy during this period. The absence of standardized vector control measures was considered representative of natural exposure conditions in the study area.

All samples were obtained as part of routine clinical practice, and no procedures were performed specifically for research purposes. Blood collection was carried out by licensed veterinarians, required less than 3 min per animal, and involved gentle manual restraint to minimize stress. All samples and associated data were anonymized before analysis. According to institutional and national regulations, and in line with European Directive 2010/63/EU on the protection of animals used for scientific purposes, formal ethical committee approval was not required for the use of surplus clinical samples not affecting patient management.

For each dog, 10 ml of blood was obtained by jugular venipuncture and divided into two tubes, one with EDTA to perform DNA extraction, and another without anticoagulant for serum separation. The tubes were maintained at room temperature. Serum samples were obtained by centrifuging the non-anticoagulated blood at 3000 rpm for 10 min. The separated serum was transferred to cryotubes and stored at −80 °C until infection and cytokine determination.

### Serological analysis

Serum samples were analyzed to detect antibodies against *A. phagocytophilum*, *E. canis*, *L. infantum*, *Leptospira* spp., and *Rickettsia* spp. using pathogen-specific serological methods.

Antibodies against *A. phagocytophylum* were detected by indirect immunofluorescent assay (IFAT) using antigen-coated slides (Fuller Laboratories), and titers > 1:40 were considered positive. Detection of anti-*E. canis* antibodies were performed by IFAT following the protocol described by [28]. The presence of antibodies against *Leptospira* spp. was determined using the microscopic agglutination test (MAT), which is considered the serological reference standard (gold standard) for diagnosis of leptospirosis owing to its high specificity in detecting anti-*Leptospira* antibodies [29]. The MAT was performed according to the standard procedures recommended by the World Organisation for Animal Health (WOAH, formerly OIE). A panel of live reference serovars representing the main pathogenic serogroups circulating in the region was used as antigens. Serum samples were initially diluted 1:50 in phosphate-buffered saline (PBS), mixed with an equal volume of each live antigen, and incubated at 28–30 °C for 1 h. Agglutination was assessed by dark-field microscopy, and samples showing ≥ 50% agglutination were considered positive. Positive sera were further titrated by serial two-fold dilutions, with titers ≥ 1:100 considered positive.

Anti-*L. infantum* immunoglobulin G (IgG) antibodies were detected using an in-house IFAT in accordance with the World Organisation for Animal Health (WOAH, formerly OIE) [30], and titers ≥ 1:80 were considered positive. Anti-*Rickettsia* spp. IgG antibodies were detected using a commercial canine IFAT kit (AffiVET/AffiGEN), applying a positivity threshold of ≥ 1:128, following the manufacturer’s instructions.

### Cytokine quantification

Serum levels of IL-2, IL-6, IL-8 (CXCL8), IL-10, IL-12/IL-23p40, nerve growth factor (NGF)-β, IFN-γ, tumor necrosis factor (TNF)-α, MCP-1 (CCL2), vascular endothelial growth factor (VEGF)-α, and SCF were quantified using the ProcartaPlex™ Canine Cytokine/Chemokine/Growth Factor Panel (ThermoFisher, Scientific, Waltham, MA, USA). Analyses were performed using the Luminex™ 200X detection system in serum samples, following the manufacturer’s instructions. All samples were analyzed in duplicate, and intra-assay and inter-assay coefficients of variation were < 10% and < 15%, respectively. Cytokine values below the detection limit were replaced with half of the minimum detectable concentration.

### Statistical analysis

Statistical analyses were performed using SAS software (version 9.2, North Carolina State University, USA). Cytokine concentrations were log_10_-transformed before analysis to reduce skewness. Linear mixed-effects models (PROC MIXED, REML estimation) were applied to evaluate the effects of breed, age group, sex, and serological status for each pathogen as fixed effects. Pairwise interaction terms between breed and age, and between breed and infection status, were specified a priori on the basis of biological plausibility and study objectives and were tested irrespective of the statistical significance of the corresponding main effects. No random effects were specified, as each dog contributed a single observation. Model assumptions were assessed through inspection of residuals. *P*-values were adjusted for multiple testing across cytokines using the Benjamini–Hochberg false discovery rate (FDR), with adjusted *q*-values < 0.05 considered statistically significant.

Descriptive statistics (mean ± SD, median, range) were calculated for all cytokines. Correlations among cytokine concentrations were evaluated using Spearman’s rank correlation coefficient (*ρ*), and correlation matrices were visualized as color-coded heatmaps with labeled *P*-values.

Principal component analysis (PCA) was performed using MetaboAnalyst on autoscaled cytokine concentrations only. Categorical variables (breed, age group, infection status) were used exclusively for sample annotation and visualization and were not included in the PCA computation. The quality of the dataset was assessed to identify potential outliers and evaluate differences among the experimental groups. Before any analysis, all datasets were preprocessed using autoscaling, followed by normalization to the median [31].

## Results

### Study population and serological findings

Of the 59 dogs included in the study, 15 were males (25.4%), and 44 were females (74.6%). According to age, 8 dogs were classified as puppies (13.6%), 12 as young (20.3%), 26 as adults (44.1%), and 13 as elders (22.0%). Overall, 27 dogs belonged to the Fonni’s breed (45.8%) and 32 were German Shepherds (54.2%) (Table 1). Serological screening revealed antibodies against *Rickettsia* spp. in 21 dogs (35.6%, 3 German Shepherd and 18 Fonni’s), *Leptospira* spp. in 6 dogs (10.2%, 3 German Shepherd and 3 Fonni‘s), *E. canis* in 3 Fonni’s dogs (5.1%), and *L. infantum* in 3 dogs (5.1%, 2 Fonni’s and one German Shepherd). No dogs were seropositive for *A. phagocytophilum* (Table 1). Serum cytokine and growth factor concentrations exhibited wide inter-individual variability. Median values were lowest for IFN-γ and highest for IL-8 and IL-12 (Table 2); a purely descriptive observation reflecting the different concentration ranges of the analytes. After log_10_ transformation, residual inspection indicated an acceptable model fit for all analytes.

**Table 1:** Epidemiological data of dogs included in the study

Variable	Categories	No. of dogs (%)
Sex	Male	15 (25.4%)
	Female	44 (74.6%)
Age	Puppy (2–12 months)	8 (13.6%)
	Young (1–2 years)	12 (20.3%)
	Adults (3–8 years)	26 (44.1%)
	Elder (9–11 years)	13 (22.0%)
Breed	Fonni’s	27 (45.8%)
	German Shepherd	32 (54.2%)
*A. phagocytophylum*	Positive	0 (0%)
	Negative	59 (100%)
*E. canis*	Positive (3 F)	3 (5.1%)
	Negative	56 (94.9%)
*Leptospira* spp.	Positive (3 F + 3 GS)	6 (10.2%)
	Negative	53 (89.8%)
*L. infantum*	Positive (2 F + 1 GS)	3 (5.1%)
	Negative	56 (94.9%)
*Rickettsia* spp.	Positive (18 F + 3 GS)	21 (35.6%)
	Negative	38 (64.4%)

*F* Fonni’s, *GS* German Shepherd

**Table 2:** Mean and standard deviation of all cytokines analyzed

	IL-2 (pg/mL)	IL-6 (pg/mL)	IL-8 (pg/mL)	IL-10 (pg/mL)	IL-12 (pg/mL)	NGF-β (pg/mL)	IFN-γ (pg/mL)	TNF-α (pg/mL)	MCP-1 (pg/mL)	VEGF-α (pg/mL)	SCF (pg/mL)
Mean	146.7	299.7	1200.9	53.6	1197.6	79.3	7.7	33.2	56.5	26.5	201.1
Standard deviation	313.7	743.7	752.8	90.4	2421.5	180.7	11.6	93.7	59.7	21.4	326.5

*IL* interleukin, *NGF* nerve growth factor, *IFN* interferon, *TNF* tumor necrosis factor, *MCP* monocyte chemoattractant protein, *VEGF* vascular endothelial growth factor, *SCF* stem cell factor. Cytokine serum levels are shown as mean ± standard deviation (SD)

Regarding fixed effects, no statistical differences were found between males and females. Age exerted a significant effect on four cytokines (IL-10, NGF-β, MCP-1, and VEGF-α), whereas breed was associated with significant differences in five cytokines (IL-10, NGF-β, IFN-γ, TNF-α, and VEGF-α; Table 3). IL-10 and NGF-β concentrations were highest in young dogs, whereas MCP-1 increased progressively with age, reaching the highest values in adult and elder animals (*q* < 0.05, FDR-adjusted). VEGF-α also varied significantly across age groups, with higher concentrations in young and elder dogs compared with puppies. Marked breed-associated differences were observed (Table 3). Fonni’s dogs exhibited significantly higher serum concentrations of IL-10 (99.1 ± 115.3 pg/mL), NGF-β (146.2 ± 227.4 pg/mL), IFN-γ (11.2 ± 13.7 pg/mL), TNF-α (49.4 ± 94.3 pg/mL), and VEGF-α (37.3 ± 26.2 pg/mL) than German Shepherd dogs (15.1 ± 28.4 pg/mL, 22.8 ± 102.4 pg/mL, 4.8 ± 8.8 pg/mL, 19.5 ± 92.4 pg/mL, and 17.4 ± 9.9 pg/mL, respectively). No sex-, age-, or breed-related differences were detected for IL-2, IL-6, IL-8, IL-12, or SCF.

**Table 3:** Cytokine serum levels according to the variables

Variable	Categories	IL-2 (pg/mL)	IL-6 (pg/mL)	IL-8 (pg/mL)	IL-10 (pg/mL)	IL-12 (pg/mL)	NGF-β (pg/mL)	IFN-γ (pg/mL)	TNF-α (pg/mL)	MCP-1 (pg/mL)	VEGF-α (pg/mL)	SCF (pg/mL)
Sex	Male	155.3 ± 330.6	228.0 ± 505.3	1174.7 ± 524.3	38.9 ± 48.3	862.4 ± 1165.3	99.2 ± 169.4	7.1 ± 11.4	52.8 ± 133.6	39.3 ± 21.4	21.8 ± 13.6	159.6 ± 181.4
	Female	121.3 ± 266.8	324.2 ± 812.7	1209.9 ± 821.3	58.6 ± 100.8	1311.8 ± 2722.9	72.5 ± 185.8	7.9 ± 11.8	26.5 ± 76.5	62.3 ± 67.2	28.1 ± 23.4	215.3 ± 363.7
*q*-value (FDR-adjusted)		0.69205	0.59409	0.84889	0.32037	0.38119	0.6112	0.8354	0.47942	0.05029	0.20736	0.4434
Age	Puppy	17.1 ± 20.4	37.9 ± 47.9	1025.1 ± 705.1	5.0 ± 37.9^a^	542.2 ± 295.1	24.4 ± 57.6^a^	6.0 ± 9.0	12.9 ± 28.8	36.6 ± 26.7^a^	18.3 ± 17.2^a^	79.5 ± 55.2
	Young	135.5 ± 177.3	213.4 ± 240.2	1214.2 ± 1226.6	116.3 ± 107.3^b^	890.4 ± 595.7	149.0 ± 186.8^b^	15.7 ± 18.0	45.0 ± 88.1	38.7 ± 25.2^a^	37.4 ± 25.9^b^	172.3 ± 135.7
	Adult	138.4 ± 325.9	257.9 ± 633.9	1175.3 ± 565.6	26.3 ± 33.8^a^	907.9 ± 1256.9	52.4 ± 128.5^a^	5.2 ± 8.8	31.2 ± 103.9	50.8 ± 52.2^b^	18.4 ± 9.6^a^	195.1 ± 349.3
	Elder	253.3 ± 449.0	624.2 ± 1269.2	1348.1 ± 597.8	67.87 ± 139.4^b^	2463.7 ± 4730.2	102.6 ± 283.6^b^	6.2 ± 8.1	38.8 ± 108.9	96.5 ± 90.2^b^	37.8 ± 28.2^b^	314.6 ± 466.6
*q*-value (FDR-adjusted)		0.41564	0.29959	0.81737	**0.0217**	0.195580	** < 0.001**	0.05972	0.89536	**0.041038**	**0.00613**	0.43536
Breed	Fonni’s	196.1 ± 329.4	419.0 ± 897.9	1276.7 ± 917.9	99.1 ± 115.3	1652.1 ± 3331.7	146.2 ± 227.4	11.2 ± 13.7	49.4 ± 94.3	56.6 ± 72.4	37.3 ± 26.2	244.8 ± 334.8
	German Shepherd	104.9 ± 298.7	199.1 ± 579.4	1137.1 ± 586.8	15.1 ± 28.4	814.0 ± 1152.9	22.8 ± 102.4	4.8 ± 8.8	19.5 ± 92.4	56.3 ± 47.6	17.4 ± 9.9	164.3 ± 319.9
*q*-value (FDR-adjusted)		0.27439	0.27967	0.49924	**0.00093**	0.22215	**0.0134**	**0.04204**	**0.0233**	0.9853	**0.00075**	0.35177

Bold numbers show statistical differences (*q* < 0.05, Benjamini–Hochberg FDR-adjusted) in cytokine serum levels. Different superscripts in the same row indicate statistical differences. *P*-values correspond to linear mixed-effects models on log-transformed data with Benjamini–Hochberg FDR correction. *IL* interleukin, *NGF* nerve growth factor, *IFN* interferon, *TNF* tumor necrosis factor, *MCP* monocyte chemoattractant protein, *VEGF* vascular endothelial growth factor, *SCF* stem cell factor. Cytokine serum levels are shown as mean ± standard deviation (SD)

### Association between infection status and cytokine profiles

**Fig. 1:**
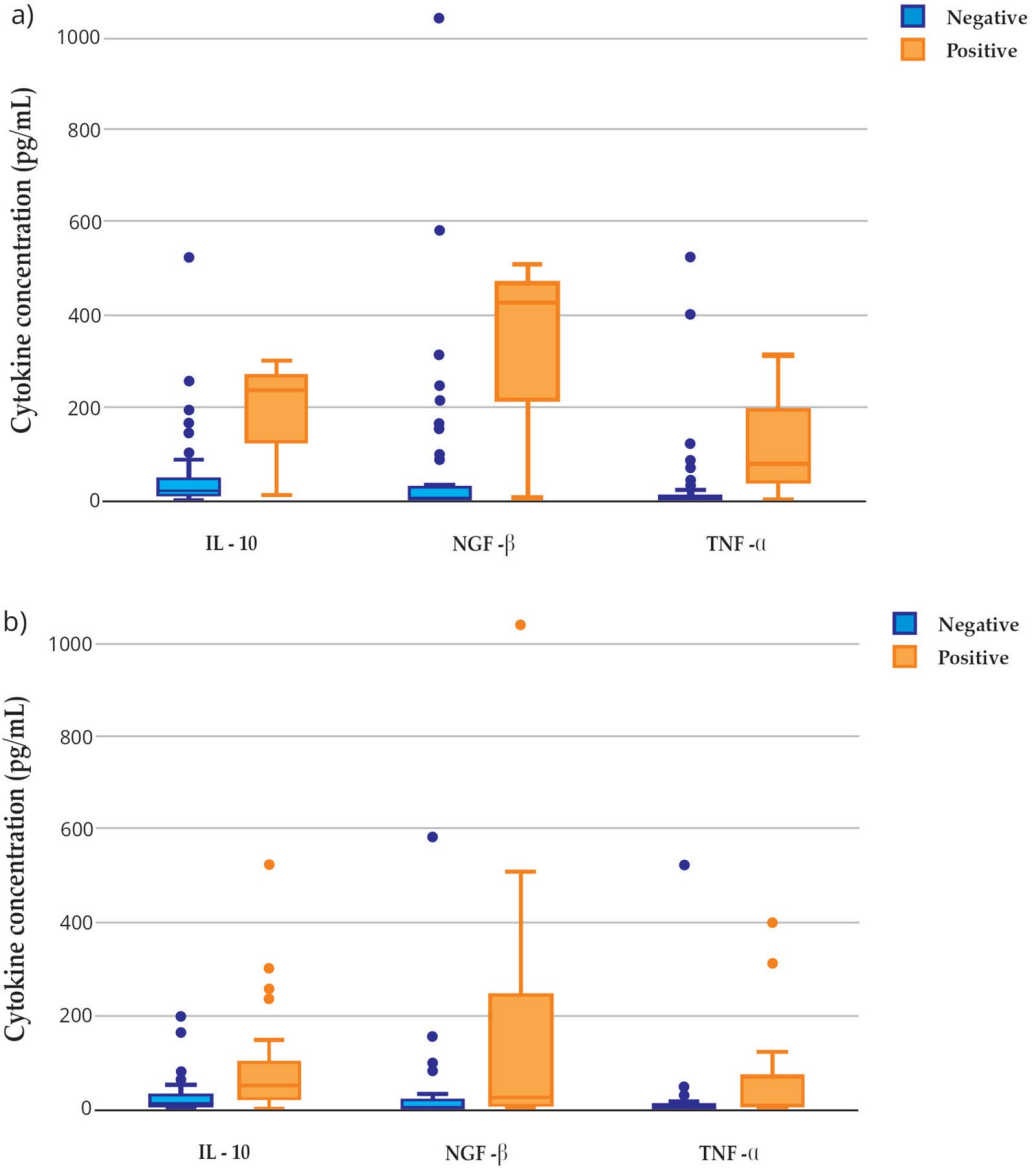
Cytokine concentrations in *L. infantum* (**a**) and *Rickettsia* spp. (**b**) positive (3 and 21, respectively) and negative (53 and 38, respectively) dogs. Boxplots show the values of cytokines statistically different between the two groups (IL-10, NGF-β, and TNF-α). *L. infantum* and *Rickettsia* spp. positive animals presented significantly higher IL-10, NGF-β, and TNF-α values compared with uninfected animals (*q* < 0.05, Benjamini–Hochberg FDR-adjusted). Results involving *L. infantum* seropositive dogs should be interpreted with caution owing to the low number of positive animals (*n* = 3). *IL* interleukin, *NGF* nerve growth factor, *TNF* tumor necrosis factor

Owing to the absence of seropositive animals, *A. phagocytophilum* was excluded from further analysis. Seropositivity for *E. canis* and *Leptospira* spp. was not associated with significant changes in cytokine concentrations after FDR correction. Dogs seropositive for *L. infantum* (Fig. 1a) and *Rickettsia* spp. (Fig. 1b) showed higher serum concentrations of IL-10, NGF-β, and TNF-α compared with seronegative animals (*q* < 0.05, FDR-adjusted). Owing to the limited number of seropositive dogs, especially for *L. infantum*, analyses were conducted considering the whole population irrespective of breed, and results should be interpreted with caution.

### Interaction effects between breed, age, and infection status

**Fig. 2:**
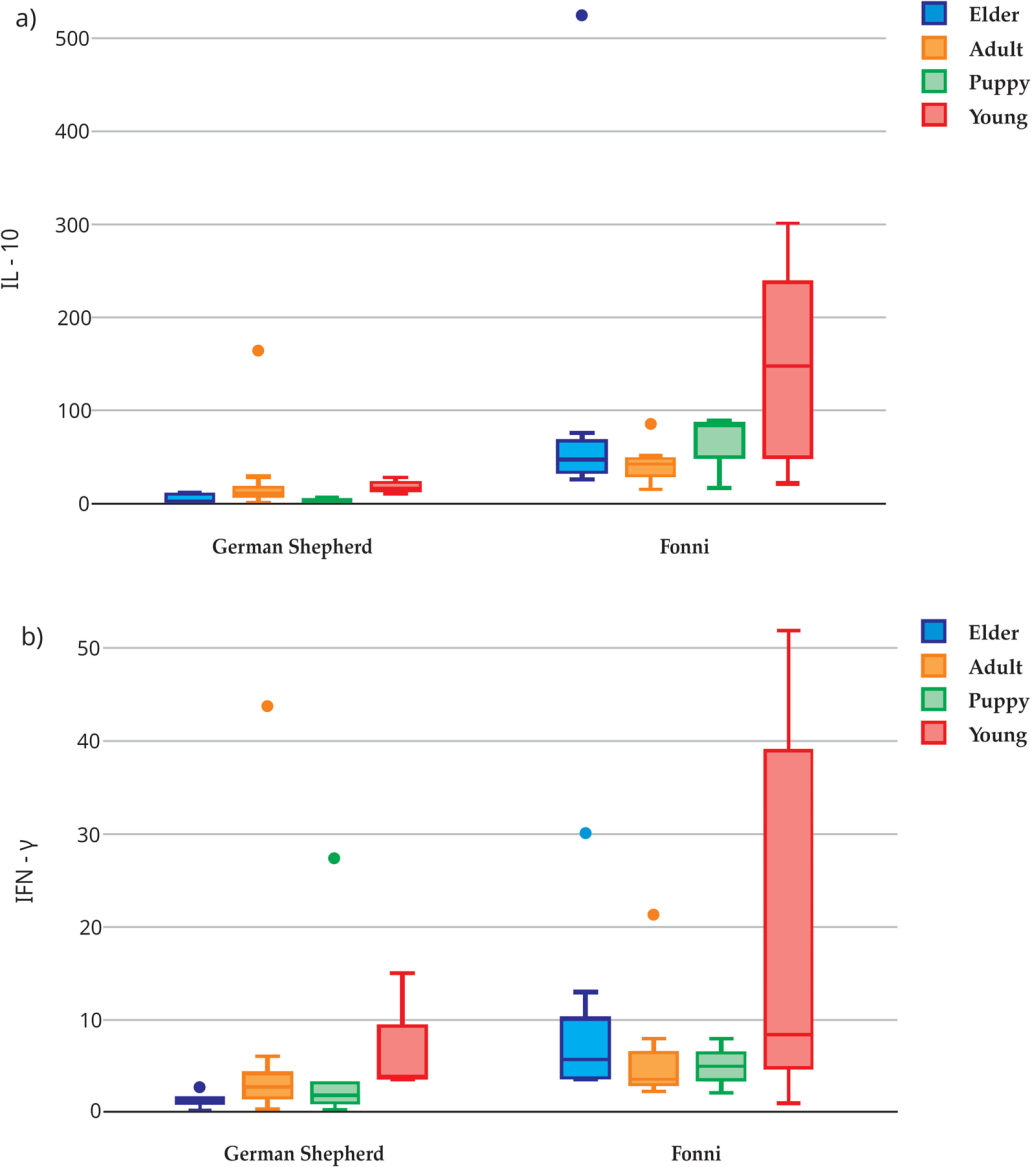
IL-10 (**a**) and IFN-γ (**b**) serum levels according to age group and breed. Boxplots show the values of cytokines statistically different between the groups. Young animals of the Fonni’s breed presented the highest values of these two cytokines (*q* < 0.05, Benjamini–Hochberg FDR-adjusted). Results involving *L. infantum*-seropositive dogs should be interpreted with caution owing to the low number of positive animals (*n* = 3). *IL* interleukin, *IFN* interferon. Number of animals included by category: 27 Fonni’s (1 elder, 6 adults, 10 puppies, and 10 young) and 24 German Shepherd (3 elders, 15 adults, 4 puppies, and 2 young)

**Fig. 3:**
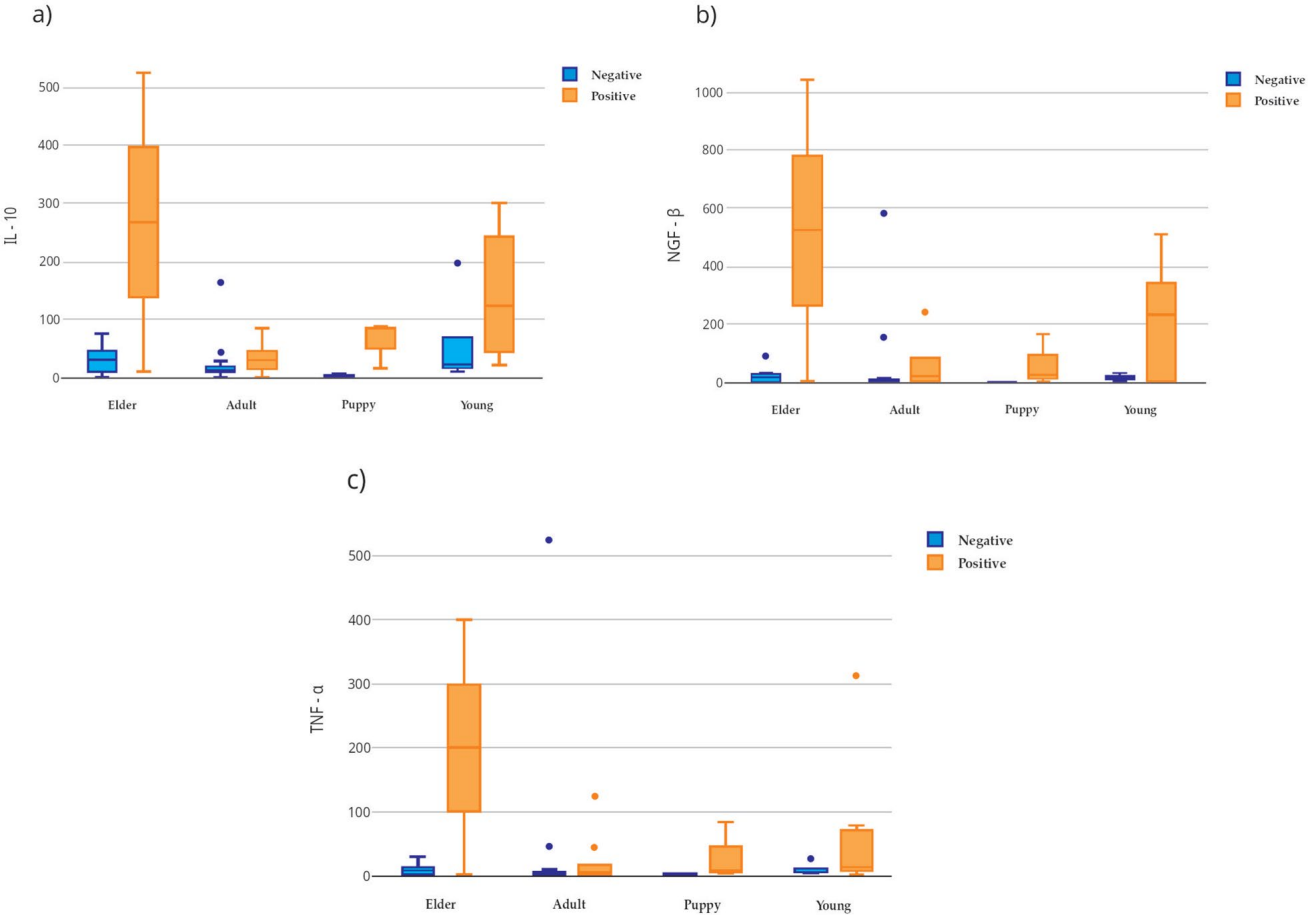
IL-10 (**a**), NGF-β (**b**), and TNF-α (**c**) serum levels according to age group in infected (positive) and uninfected (negative) *Rickettsia* spp. dogs. Boxplots show the values of cytokines statistically different between the groups. Elder infected dogs presented the highest values of these three cytokines (*q* < 0.05, Benjamini–Hochberg FDR-adjusted). *IL* interleukin, *NGF* nerve growth factor, *TNF* tumor necrosis factor. Number of animals included by category: 20 infected (2 elders, 6 adults, 3 puppies, and 9 young) and 24 uninfected (2 elders, 15 adults, 11 puppies, and 3 young)

The pairwise interaction analysis revealed a significant interaction between breed and age for IL-10 and IFN-γ concentrations. For IFN-γ, although no overall age-related main effect was detected, the breed-associated differences varied across age groups, resulting in a significant breed × age interaction. Young Fonni’s dogs displayed higher levels of both cytokines compared with age-matched German Shepherds (*q* < 0.05, FDR-adjusted) (Fig. 2). In addition, an interaction between age and *Rickettsia* spp. seropositivity was observed for IL-10, NGF-β, and TNF-α, with the highest concentrations detected in seropositive elder dogs (*q* < 0.05, FDR-adjusted) (Fig. 3).

### Cytokine correlation analysis

**Fig. 4:**
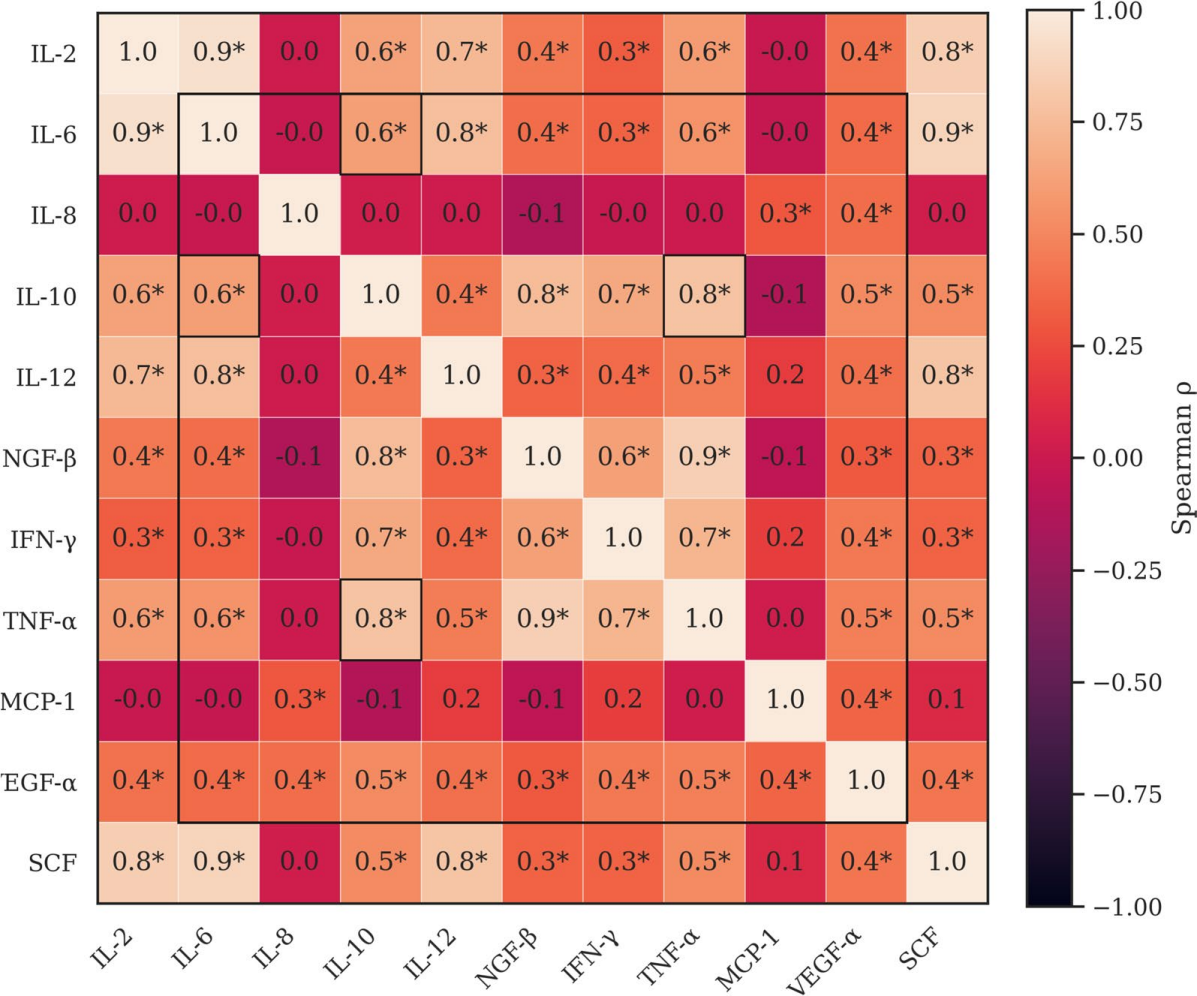
Spearman correlation matrix among cytokines. Color intensity indicates the strength and direction of Spearman correlations (*ρ*) among cytokines. Asterisks denote statistically significant correlations after Benjamini–Hochberg false discovery rate (FDR) correction (*q*-value < 0.05)

Figure 4 displays pairwise Spearman correlation coefficients (*ρ*) among serum cytokine values. A significant positive correlation cluster involving IL-6, TNF-α, MCP-1, and VEGF-α (*ρ* = 0.56–0.78, *q* < 0.05, FDR-adjusted) is indicative of coordinated pro-inflammatory and angiogenic responses. IL-10 showed weak or context-dependent associations with pro-inflammatory cytokines, consistent with a regulatory role.

### Multivariate analysis of cytokine profiles

**Fig. 5:**
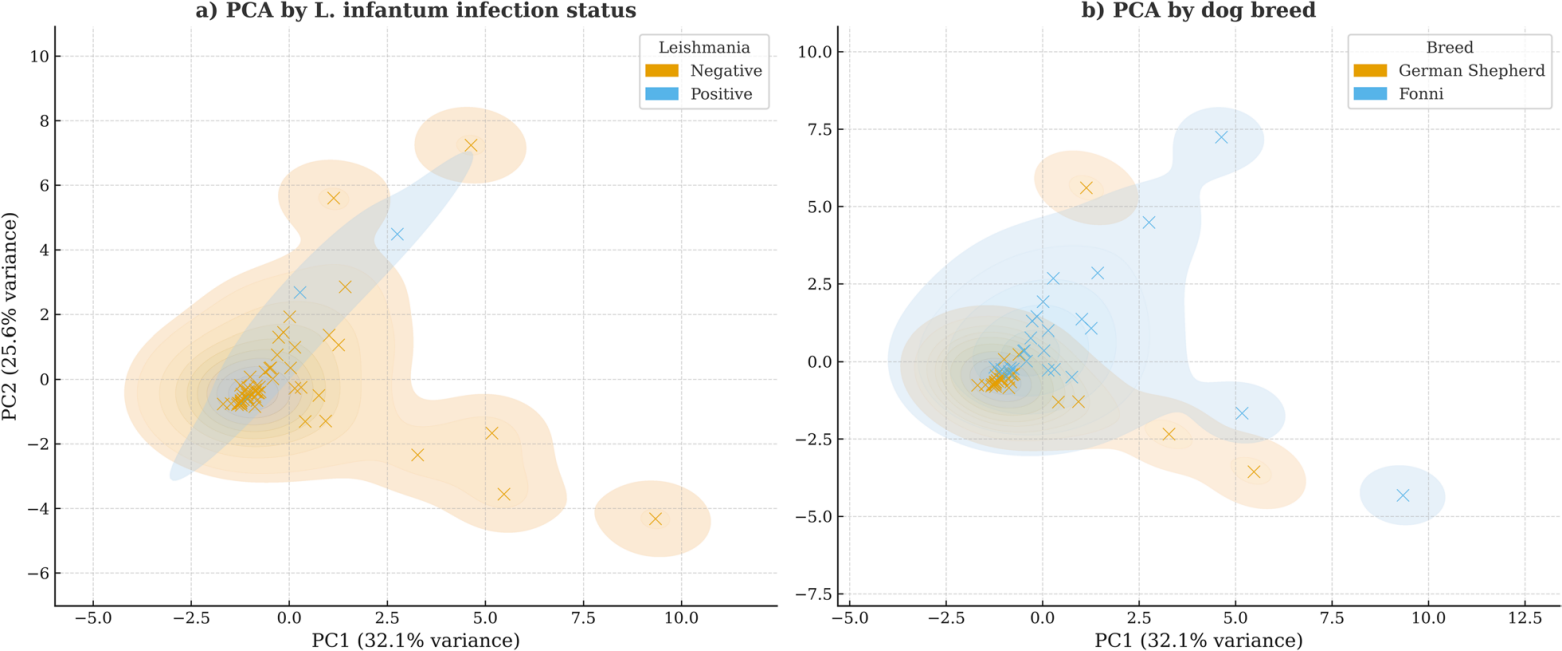
Principal component analysis (PCA) of cytokine and immune factor profiles. PCA was conducted using standardized concentrations of 11 cytokines (IL-2, IL-6, IL-8, IL-10, IL-12, NGF-β, IFN-γ, TNF-α, MCP-1, VEGF-α, and SCF) only. Categorical variables (breed and infection status) were used exclusively for visualization and were not included in the PCA computation. Each point represents an individual dog, while shaded areas indicate the distribution of samples within each group. (**a**) Cluster according to *L. infantum* infection status, and (**b**) cluster according to dog breed. Given the low number of *L. infantum*-seropositive dogs (*n* = 3), clustering by infection status should be interpreted cautiously. *IL* interleukin, *NGF* nerve growth factor, *IFN* interferon, *TNF* tumor necrosis factor, *MCP* monocyte chemoattractant protein, *VEGF* vascular endothelial growth factor, *SCF* stem cell factor

PCA performed on autoscaled cytokine concentrations showed that the first two components explained 57.7% of total variance (PC1 = 32.1% and PC2 = 25.6%). PC1 was mainly driven by proinflammatory and proliferative cytokines (IL-2, IL-6, IL-12, and SCF), whereas PC2 was associated with regulatory and modulatory responses (NGF-β, IL-10, TNF-α, and IFN-γ). Visualization of PCA scores indicated partial separation according to breed and *L. infantum* serological status (Fig. 5a), with Fonni’s dogs clustering toward higher PC2 values (Fig. 5b).

## Discussion

This study provides evidence that prolonged historical exposure, occurring over centuries to millennia under Mediterranean endemic conditions, to vector-borne pathogens can shape breed-specific immune regulation in dogs, with the Fonni’s breed displaying a profile consistent with infection tolerance rather than inflammatory susceptibility. This observation does not imply direct genetic co-evolution over a defined evolutionary timescale, but rather long-standing exposure across multiple generations under stable endemic pressure. A limitation of this study is the observational design and the use of privately owned dogs, which may introduce variability in environmental exposure and husbandry conditions. The cytokine panel revealed wide inter-individual variability, yet consistent breed-related patterns emerged. Fonni’s dogs displayed significantly higher serum levels of IL-10, NGF-β, IFN-γ, TNF-α, and VEGF-α compared with German Shepherds, while MCP-1 was more elevated in adult and elder dogs of both breeds. These results highlight a distinctive immunological profile in the Fonni’s breed, which is consistent with previous reports suggesting that Mediterranean native breeds exposed to long-standing endemic vector-borne pathogens may display immune regulatory patterns compatible with partial resistance or tolerance to *L. infantum* infection [32]. However, German Shepherds originated in a temperate European environment with different ecological and vector-borne disease pressure and therefore should be regarded as a comparative population rather than an evolutionary control. Consequently, the observed differences may reflect contrasting historical exposure to vector-borne pathogens rather than exclusively intrinsic breed-specific adaptations.

No significant differences were detected between sexes, while age exerted significant effects on IL-10, NGF-β, MCP-1, and VEGF-α concentrations. Young Fonni’s dogs displayed the highest IL-10 and NGF-β levels, whereas adults and elders showed increased MCP-1. These findings agreed with studies reporting Th2-polarized responses in immature immune systems [33–35] and inflammation-associated cytokine increases with aging [36, 37]. Dogs seropositive for *L. infantum* and *Rickettsia* spp. exhibited significantly higher IL-10, NGF-β, and TNF-α levels than uninfected animals. These cytokines are often upregulated during chronic or subclinical infections, where they act to control inflammation [38–40]. Considering these findings, both *Leishmania* and *Rickettsia* spp. seropositives were associated with increased IL-10, NGF-β, and TNF-α concentrations, suggesting overlapping immune regulatory responses to chronic or repeated vector-borne pathogen exposure. However, *L. infantum* remains epidemiologically and biologically distinct as a persistent intracellular protozoan and the main zoonotic pathogen in the Mediterranean basin, whereas *Rickettsia* spp. are associated with transient bacterial infections. Thus, similarities probably result from common regulatory pathways rather than the same immunopathogenic processes.

The constitutively elevated IL-10 and NGF-β levels observed in Fonni’s dogs, independent of infection status, suggest a preexisting regulatory immune phenotype rather than a purely infection-driven response. IL-10 is a potent immunomodulatory cytokine that downregulates Th1-driven inflammation and macrophage activation, thereby reducing tissue damage [18, 41, 42]. NGF-β is increasingly recognized as a regulator of macrophage oxidative activity and immune cell survival [43, 44]. Experimental models show that NGF-β enhances macrophage hydrogen peroxide production and inhibits *L. donovani* replication [45], suggesting a dual role in both parasite control and inflammation limitation. Together with elevated IL-10, this may constitute a tolerance-based immune strategy compatible with a regulatory immune environment that may limit immunopathology under endemic exposure.

Such tolerance-based immune strategies have been described in other host–parasite systems and may represent an evolutionarily stable response in an endemic setting, where limiting immunopathology is as critical as controlling parasite replication [46–48]. Fonni’s dogs also exhibited higher IFN-γ levels than German Shepherds, particularly in young animals. This cytokine is central to Th1 polarization and macrophage activation [49–52]. Their co-existence with elevated IL-10 suggests a balanced immune regulation that can control intracellular pathogens while minimizing collateral tissue damage. Such an equilibrium has been proposed in other naturally resistant breeds such as the Ibizan hound [32].

IL-6, TNF-α, and MCP-1 formed a correlated pro-inflammatory cluster, as confirmed by Spearman analysis. This cluster likely reflects a coordinated response involving monocyte recruitment and vascular remodeling. VEGF-α, which promotes endothelial permeability and angiogenesis, also correlated with IL-6 and TNF-α, reinforcing its contribution to leukocyte trafficking and tissue repair [53, 54]. Fonni’s dog’s higher VEGF-α levels may therefore support rapid immune cell recruitment without inducing excessive inflammation.

The PCA confirmed that cytokine profiles distinguished both infected and uninfected dogs and separated Fonni’s from German Shepherds along principal components dominated by pro-inflammatory and anti-inflammatory cytokines. This statistical clustering supports the hypothesis that Fonni’s dogs maintain a distinct immune signature, probably shaped by long-term exposure to Sardinian eco-epidemiological conditions [24, 55, 56].

Taken together, the Fonni’s dog’s cytokine pattern (elevated IL-10, NGF-β, IFN-γ, TNF-α, and VEGF-α) supports the existence of an evolved immune tolerance mechanism that is compatible with a regulatory immune environment that may limit immunopathology in endemic settings. Similar adaptive strategies have been proposed by Llobat and colleagues in other Mediterranean breeds exposed to endemic pathogens, highlighting the influence of genetic background and environmental selection on canine immune profiles [22, 32, 57]. The combination of regulatory (IL-10, NGF-β) and protective (IL-12, IFN-γ) cytokines indicates a finely tuned immune equilibrium that could be harnessed for vaccine development or immunomodulatory therapies aimed at achieving consistent results with reduced immunopathology under endemic exposure.

## Conclusions

Fonni’s dog exhibits a distinct cytokine and growth factor signature characterized by elevated IL-10, NGF-β, IFN-γ, TNF-α, and VEGF-α concentrations, supporting a tolerant immune strategy against *L. infantum* infection. Fonni’s dogs appear capable of modulating their immune response in a manner compatible with limited immunopathology under endemic exposure.

Overall, these findings reinforce previous evidence that breed-specific immune adaptations strongly influence the outcome of *L. infantum* infection in dogs. Understanding these natural resistance mechanisms in native breeds such as Fonni’s dogs may offer valuable models for developing sustainable control strategies for zoonotic leishmaniosis, including the identification of immune biomarkers associated with tolerance, the design of immunomodulatory or vaccine approaches aimed at limiting immunopathology rather than inducing sterilizing immunity, and the use of naturally tolerant breeds as epidemiological models to inform integrated disease control in endemic settings.

## Data Availability

Data supporting the main conclusions of this study are included in the manuscript.

## References

[1] Kostopoulou D, Gizzarelli M, Ligda P, Foglia Manzillo V, Saratsi K, Montagnaro S, et al. Mapping the canine vector-borne disease risk in a Mediterranean area. Parasit Vectors. 2020;13:282. 10.1186/s13071-020-04153-8. 32493470 10.1186/s13071-020-04153-8 PMC7268178

[2] Montoya-Alonso JA, Morchón R, Costa-Rodríguez N, Matos JI, Falcón-Cordón Y, Carretón E. Current distribution of selected vector-borne diseases in dogs in Spain. Front Vet Sci. 2020;7:564429. 10.3389/fvets.2020.564429. 33195540 10.3389/fvets.2020.564429 PMC7643126

[3] Costa CHN, Chang K-P, Costa DL, Cunha FVM. From infection to death: an overview of the pathogenesis of visceral leishmaniasis. Pathogens. 2023;12:969. 10.3390/pathogens12070969. 37513817 10.3390/pathogens12070969 PMC10384967

[4] Alvar J, Vélez ID, Bern C, Herrero M, Desjeux P, Cano J, et al. Leishmaniasis worldwide and global estimates of its incidence. PLoS ONE. 2012;7:e35671. 10.1371/journal.pone.0035671. 22693548 10.1371/journal.pone.0035671 PMC3365071

[5] Steverding D. The history of leishmaniasis. Parasit Vectors. 2017;10:82. 10.1186/s13071-017-2028-5. 28202044 10.1186/s13071-017-2028-5 PMC5312593

[6] Sadlova J, Hoskova A, Vojtkova B, Becvar T, Volf P. The development of *L. major*, *L. donovani* and *L. martiniquensis*, *Leishmania* currently emerging in Europe, in the sand fly species *Phlebotomus perniciosus* and *P. tobbi*. PLoS Negl Trop Dis. 2024;18:e0012597. 10.1371/journal.pntd.0012597. 39405300 10.1371/journal.pntd.0012597 PMC11508123

[7] Ready PD. Epidemiology of visceral leishmaniasis. Clin Epidemiol. 2014;6:147–54. 10.2147/CLEP.S44267. 24833919 10.2147/CLEP.S44267 PMC4014360

[8] Torres-Guerrero E, Quintanilla-Cedillo MR, Ruiz-Esmenjaud J, Arenas R. Leishmaniasis: a review. F1000Res. 2017;6:750. 10.12688/f1000research.11120.1. 28649370 10.12688/f1000research.11120.1 PMC5464238

[9] Pasquier G, Demar M, Lami P, Zribi A, Marty P, Buffet P, et al. Leishmaniasis epidemiology in endemic areas of metropolitan France and its overseas territories from 1998 to 2020. PLoS Negl Trop Dis. 2022;16:e0010745. 10.1371/journal.pntd.0010745. 36206322 10.1371/journal.pntd.0010745 PMC9624409

[10] Montaner-Angoiti E, Llobat L. Is leishmaniasis the new emerging zoonosis in the world? Vet Res Commun. 2023 Dec;47(4):1777-1799. 10.1007/s11259-023-10171-5. 37438495 10.1007/s11259-023-10171-5

[11] Schäfer I, Volkmann M, Beelitz P, Merle R, Müller E, Kohn B. Retrospective evaluation of vector-borne infections in dogs imported from the Mediterranean region and southeastern Europe (2007–2015). Parasit Vectors. 2019;12:30. 10.1186/s13071-018-3284-8. 30635034 10.1186/s13071-018-3284-8 PMC6330426

[12] Colombo M, Morelli S, Simonato G, Di Cesare A, Veronesi F, di Frangipane Regalbono A, et al. Exposure to major vector-borne diseases in dogs subjected to different preventative regimens in endemic areas of Italy. Pathogens. 2021;10:507. 10.3390/pathogens10050507. 33922459 10.3390/pathogens10050507 PMC8146437

[13] Piredda I, Ponti MN, Piras A, Palmas B, Pintore P, Pedditzi A, et al. New insights on *Leptospira* infections in a canine population from North Sardinia, Italy: a sero-epidemiological study. Biology. 2021;10:507. 10.3390/biology10060507. 34200298 10.3390/biology10060507 PMC8226461

[14] Cocco R, Sechi S, Marín-García PJ, Liotta L, Llobat L. Seroprevalence of zoonotic pathogens and related haematological and biochemical profiles in Fonni’s dogs in rural conditions. Vet Microbiol. 2025;305:110540. 10.1016/j.vetmic.2025.110540. 40339256 10.1016/j.vetmic.2025.110540

[15] Baxarias M, Álvarez-Fernández A, Martínez-Orellana P, Montserrat-Sangrà S, Ordeix L, Rojas A, et al. Does co-infection with vector-borne pathogens play a role in clinical canine leishmaniosis? Parasit Vectors. 2018;11:135. 10.1186/s13071-018-2724-9. 29554918 10.1186/s13071-018-2724-9 PMC5859550

[16] Beasley EA, Pessôa-Pereira D, Scorza BM, Petersen CA. Epidemiologic, clinical and immunological consequences of co-infections during canine leishmaniosis. Animals. 2021;11:3206. 10.3390/ani11113206. 34827938 10.3390/ani11113206 PMC8614518

[17] Strauss-Ayali D, Baneth G, Shor S, Okano F, Jaffe CL. Interleukin-12 augments a Th1-type immune response manifested as lymphocyte proliferation and interferon gamma production in *Leishmania infantum*-infected dogs. Int J Parasitol. 2005;35:63–73. 10.1016/j.ijpara.2004.10.015. 15619517 10.1016/j.ijpara.2004.10.015

[18] Solano-Gallego L, Montserrrat-Sangrà S, Ordeix L, Martínez-Orellana P. *Leishmania**infantum*-specific production of IFN-γ and IL-10 in stimulated blood from dogs with clinical leishmaniosis. Parasit Vectors. 2016;9:317. 10.1186/s13071-016-1598-y. 27260142 10.1186/s13071-016-1598-y PMC4893235

[19] Maia C, Campino L. Biomarkers Associated With Leishmania infantum Exposure, Infection, and Disease in Dogs. Front Cell Infect Microbiol. 2018 Sep 6;8:302. 10.3389/fcimb.2018.00302. 30237985 10.3389/fcimb.2018.00302 PMC6136405

[20] Ribeiro FN, de Souza TL, Menezes RC, Keidel L, dos Santos JPR, da Silva IJ, et al. Anatomical vascular differences and *Leishmania*-induced vascular morphological changes are associated with a high parasite load in the skin of dogs infected with *Leishmania**infantum*. Pathogens. 2024;13:371. 10.3390/pathogens13050371. 38787223 10.3390/pathogens13050371 PMC11123845

[21] Álvarez L, Marín-García P-J, Llobat L. Immunological and genomic characterization of Ibizan Hound dogs in an endemic *Leishmania**infantum* region. Parasit Vectors. 2022;15:445. 10.1186/s13071-022-05504-3. 36443886 10.1186/s13071-022-05504-3 PMC9706964

[22] Amato A, Cavallo C, Marín-García PJ, Emmanuele G, Tomasello M, Tomasella C, et al. Effect of breed on hematological and biochemical parameters of apparently healthy dogs infected with zoonotic pathogens endemic to the Mediterranean Basin. Animals. 2024;14:1516. 10.3390/ani14111516. 38891563 10.3390/ani14111516 PMC11171318

[23] Dreger DL, Davis BW, Cocco R, Sechi S, Di Cerbo A, Parker HG, et al. Commonalities in development of pure breeds and population isolates revealed in the genome of the Sardinian Fonni’s Dog. Genetics. 2016;204:737–55. 10.1534/genetics.116.192427. 27519604 10.1534/genetics.116.192427 PMC5068859

[24] Tamponi C, Scarpa F, Carta S, Knoll S, Sanna D, Gai C, et al. Seroprevalence and risk factors associated with *Leishmania infantum* in dogs in Sardinia (Italy), an endemic island for leishmaniasis. Parasitol Res. 2021;120:289–300. 10.1007/s00436-020-06973-0. 33205238 10.1007/s00436-020-06973-0 PMC7846507

[25] Chisu V, Tanda A, Sechi S, Pinna Parpaglia ML, Masu G, Loi F, et al. Clinical study and serological diagnosis of vector-borne pathogens in Sardinian dogs. Vet Sci. 2024;11:313. 10.3390/vetsci11070313. 39057997 10.3390/vetsci11070313 PMC11281559

[26] The ARRIVE guidelines 2.0 | ARRIVE Guidelines. https://arriveguidelines.org/arrive-guidelines.

[27] Solano-Gallego L, Miró G, Koutinas A, Cardoso L, Pennisi MG, Ferrer L, et al. LeishVet guidelines for the practical management of canine leishmaniosis. Parasit Vectors. 2011;4:86. 10.1186/1756-3305-4-86. 21599936 10.1186/1756-3305-4-86 PMC3125381

[28] Dawson JE, Anderson BE, Fishbein DB, Sanchez JL, Goldsmith CS, Wilson KH, et al. Isolation and characterization of an *Ehrlichia* sp. from a patient diagnosed with human ehrlichiosis. J Clin Microbiol. 1991;29:2741–5. 10.1128/jcm.29.12.2741-2745.1991. 1757543 10.1128/jcm.29.12.2741-2745.1991 PMC270425

[29] Goris MGA, Hartskeerl RA. Leptospirosis serodiagnosis by the microscopic agglutination test. Curr Protoc Microbiol. 2014;32:Unit 12E.5. 10.1002/9780471729259.mc12e05s32 10.1002/9780471729259.mc12e05s32 24510846

[30] Codes and Manuals [Internet]. WOAH - World Organisation for Animal Health. https://www.woah.org/en/what-we-do/standards/codes-and-manuals/.

[31] Wold S. PLS Modeling with Latent Variables in Two Or More Dimensions. 1987.

[32] Martínez-Sáez L, Marín-García PJ, Amato A, Cavallo C, Boccuni S, Tomasello M, et al. Breed-specific variation in canine cytokine profiles in a leishmaniosis-endemic region. Microb Pathog. 2025;209:108096. 10.1016/j.micpath.2025.108096. 41062003 10.1016/j.micpath.2025.108096

[33] Genovese F, Mancuso G, Cuzzola M, Biondo C, Beninati C, Delfino D, et al. Role of IL-10 in a neonatal mouse listeriosis model. J Immunol. 1999;163:2777–82. 10453021

[34] Antonelli A, Bracci-Laudiero L, Aloe L. Altered plasma nerve growth factor-like immunoreactivity and nerve growth factor-receptor expression in human old age. Gerontology. 2003;49:185–90. 10.1159/000069170. 12679610 10.1159/000069170

[35] Pereira M, Valério-Bolas A, Saraiva-Marques C, Alexandre-Pires G, da Pereira Fonseca I, Santos-Gomes G. Development of dog immune system: from in uterus to elderly. Vet Sci. 2019;6:83. 10.3390/vetsci6040083. 31640234 10.3390/vetsci6040083 PMC6958461

[36] Brüünsgaard H, Pedersen BK. Age-related inflammatory cytokines and disease. Immunol Allergy Clin North Am. 2003;23:15–39. 10.1016/s0889-8561(02)00056-5. 12645876 10.1016/s0889-8561(02)00056-5

[37] Michaud M, Balardy L, Moulis G, Gaudin C, Peyrot C, Vellas B, et al. Proinflammatory cytokines, aging, and age-related diseases. J Am Med Dir Assoc. 2013;14:877–82. 10.1016/j.jamda.2013.05.009. 23792036 10.1016/j.jamda.2013.05.009

[38] Wilson EB, Brooks DG. The role of IL-10 in regulating immunity to persistent viral infections. Curr Top Microbiol Immunol. 2011;350:39–65. 10.1007/82_2010_96. 20703965 10.1007/82_2010_96 PMC3492216

[39] Maia C, Campino L. Cytokine and phenotypic cell profiles of *Leishmania infantum* infection in the dog. J Trop Med. 2012;2012:541571. 10.1155/2012/541571. 21845197 10.1155/2012/541571 PMC3154519

[40] Ley S, Weigert A, Weichand B, Henke N, Mille-Baker B, Janssen RaJ, et al. The role of TRKA signaling in IL-10 production by apoptotic tumor cell-activated macrophages. Oncogene. 2013;32:631–40. 10.1038/onc.2012.77. 22410777 10.1038/onc.2012.77

[41] Fiorentino DF, Zlotnik A, Mosmann TR, Howard M, O’Garra A. IL-10 inhibits cytokine production by activated macrophages. J Immunol. 1991;147:3815–22. 1940369

[42] Ng THS, Britton GJ, Hill EV, Verhagen J, Burton BR, Wraith DC. Regulation of Adaptive Immunity; The Role of Interleukin-10. Front Immunol. Frontiers; 2013;4. 10.3389/fimmu.2013.00129 10.3389/fimmu.2013.00129 PMC3668291 23755052

[43] la Sa A, Corinti S, Federici M, Saragovi HU, Girolomoni G. Ligand activation of nerve growth factor receptor TrkA protects monocytes from apoptosis. J Leukoc Biol. 2000;68:104–10. 10914496

[44] Prencipe G, Minnone G, Strippoli R, De Pasquale L, Petrini S, Caiello I, et al. Nerve growth factor downregulates inflammatory response in human monocytes through TrkA. J Immunol. 2014;192:3345–54. 10.4049/jimmunol.1300825. 24585880 10.4049/jimmunol.1300825

[45] Chiba R, Amagai Y, Tanaka A, Katakura K, Matsuda H. Nerve growth factor promotes killing of *Leishmania donovani* by macrophages through the induction of hydrogen peroxide. Microbes Infect. 2014;16:702–6. 10.1016/j.micinf.2014.06.001. 24937592 10.1016/j.micinf.2014.06.001

[46] Schneider DS, Ayres JS. Two ways to survive infection: what resistance and tolerance can teach us about treating infectious diseases. Nat Rev Immunol. 2008;8:889–95. 10.1038/nri2432. 18927577 10.1038/nri2432 PMC4368196

[47] Carvalho EM, Bacellar O, Brownell C, Regis T, Coffman RL, Reed SG. Restoration of IFN-gamma production and lymphocyte proliferation in visceral leishmaniasis. J Immunol. 1994;152:5949–56. 10.4049/jimmunol.152.12.5949. 8207220

[48] Soares MP, Gozzelino R, Weis S. Tissue damage control in disease tolerance. Trends Immunol. 2014;35:483–94. 10.1016/j.it.2014.08.001. 25182198 10.1016/j.it.2014.08.001

[49] Magram J, Connaughton SE, Warrier RR, Carvajal DM, Wu CY, Ferrante J, et al. IL-12-deficient mice are defective in IFN gamma production and type 1 cytokine responses. Immunity. 1996;4:471–81. 10.1016/s1074-7613(00)80413-6. 8630732 10.1016/s1074-7613(00)80413-6

[50] Xing Z, Zganiacz A, Santosuosso M. Role of IL-12 in macrophage activation during intracellular infection: IL-12 and mycobacteria synergistically release TNF-alpha and nitric oxide from macrophages via IFN-gamma induction. J Leukoc Biol. 2000;68:897–902. 11129658

[51] Su X, Yu Y, Zhong Y, Giannopoulou EG, Hu X, Liu H, et al. Interferon-γ regulates cellular metabolism and mRNA translation to potentiate macrophage activation. Nat Immunol. 2015;16:838–49. 10.1038/ni.3205. 26147685 10.1038/ni.3205 PMC4509841

[52] Ullrich KA-M, Schulze LL, Paap E-M, Müller TM, Neurath MF, Zundler S. Immunology of IL-12: an update on functional activities and implications for disease. EXCLI J. 2020;19:1563–89. 10.17179/excli2020-3104. 33408595 10.17179/excli2020-3104 PMC7783470

[53] Sato T, Takeuchi M, Karasawa Y, Takayama K, Enoki T. Comprehensive expression patterns of inflammatory cytokines in aqueous humor of patients with neovascular age-related macular degeneration. Sci Rep. 2019;9:19447. 10.1038/s41598-019-55191-x. 31857597 10.1038/s41598-019-55191-x PMC6923359

[54] Obadă O, Pantalon AD, Rusu-Zota G, Hăisan A, Lupuşoru SI, Constantinescu D, et al. Aqueous humor cytokines in non-proliferative diabetic retinopathy. Medicina Kaunas. 2022;58:909. 10.3390/medicina58070909. 35888628 10.3390/medicina58070909 PMC9324281

[55] Cortellari M, Bionda A, Cocco R, Sechi S, Liotta L, Crepaldi P. Genomic analysis of the endangered Fonni’s dog breed: a comparison of genomic and phenotypic evaluation scores. Animals Basel. 2023;13:818. 10.3390/ani13050818. 36899675 10.3390/ani13050818 PMC10000202

[56] Cocco R, Sechi S, Rizzo M, Bonomo A, Arfuso F, Giudice E. Haematochemical profile of healthy dogs seropositive for single or multiple vector-borne pathogens. Vet Sci. 2024;11:205. 10.3390/vetsci11050205. 38787177 10.3390/vetsci11050205 PMC11126013

[57] Álvarez L, Marín-García P-J, Llobat L. Genetic haplotypes associated with immune response to *Leishmania infantum* infection in dogs. Vet Res Commun. 2023;47:1675–85. 10.1007/s11259-023-10123-z. 37059873 10.1007/s11259-023-10123-z

